# A new technology for increasing therapeutic protein levels in the brain over extended periods

**DOI:** 10.1371/journal.pone.0214404

**Published:** 2019-04-12

**Authors:** Ryosuke Nakano, Sayaka Takagi-Maeda, Yuji Ito, Satoshi Kishimoto, Tomoko Osato, Kaori Noguchi, Kana Kurihara-Suda, Nobuaki Takahashi

**Affiliations:** 1 Research and Development Division, Kyowa Hakko Kirin Co., LTD., Machida-shi, Tokyo, Japan; 2 Department of Chemistry and Bioscience, Graduate School of Science and Engineering, Kagoshima University, Kagoshima, Japan; 3 Research and Development Division, Kyowa Hakko Kirin Co., LTD., Chiyoda-ku, Tokyo, Japan; University of Pécs Medical School, HUNGARY

## Abstract

Effective delivery of protein therapeutics into the brain remains challenging because of difficulties associated with crossing the blood-brain barrier (BBB). To overcome this problem, many researchers have focused on antibodies binding the transferrin receptor (TfR), which is expressed in endothelial cells, including those of the BBB, and is involved in receptor-mediated transcytosis (RMT). RMT and anti-TfR antibodies provide a useful means of delivering therapeutics into the brain, but the anti-TfR antibody has a short half-life in blood because of its broad expression throughout the body. As a result, anti-TfR antibodies are only maintained at high concentrations in the brain for a short time. To overcome this problem, we developed a different approach which slows down the export of therapeutic antibodies from the brain by binding them to a brain-specific antigen. Here we report a new technology, named AccumuBrain, that achieves both high antibody concentration in the brain and a long half-life in blood by binding to myelin oligodendrocyte glycoprotein (MOG), which is specifically expressed in oligodendrocytes. We report that, using our technology, anti-MOG antibody levels in the brains of mice (*Mus musculus*) and rats (*Rattus norvegicus*) were increased several tens of times for a period of one month. The mechanism of this technology is different from that of RMT technologies like TfR and would constitute a breakthrough for central nervous system disease therapeutics.

## Introduction

There are many protein therapeutics, including antibodies and enzymes, undergoing clinical trials for central nerve system (CNS) diseases. It is well known that delivery of protein therapeutics into the brain remains challenging due to the difficulty of crossing the blood-brain barrier (BBB). This is illustrated by the fact that the concentration ratio of IgG in CNS relative to plasma has been reported to be in the range of 1:500 [1–5].

Therapeutic enzymes are effective against, and have been approved for, lysosomal storage diseases such as Fabry disease and Gaucher disease, but they are not effective against central nervous system conditions because their molecular weight is too high to allow for passage across the BBB [6,7].

To increase the concentration of therapeutic proteins in the brain, direct intrathecal and intracerebral injection has been tested, but the procedure is highly invasive [8]. Furthermore, it is reported that IgG are rapidly discharged from the brain into circulating blood by neonatal Fc receptor (FcRn) [9,10].

To overcome these problems, extensive research has been undertaken into technologies for delivering drugs into the brain. Most popular technologies use a receptor-mediated transcytosis (RMT)-based mechanism, and there are many reports of antibodies which bind to the transferrin receptor (TfR), which is expressed in endothelial cells, including those of the BBB, and allows for transport across the BBB by RMT [11,12]. Additionally, other receptors, such as the insulin receptor, are also reportedly transported across the BBB by means of RMT [13].

For clinical use, anti-TfR antibody or anti-insulin receptor antibody are fused with therapeutic antibodies or enzymes. One example is the anti-BACE1 anti-TfR bispecific antibody, which is transported across the BBB by RMT of TfR and has been found to be present in the brain at levels four times higher than that in control mice [14]. Other examples include anti-insulin receptor antibodies fused with glial cell-derived neurotrophic factor (GDNF) or iduronidase (IDUA). Anti-insulin receptor antibody GDNF fusion protein concentration is reported to display a 10-fold increase in the brains of Rhesus monkeys (*Macaca mulatta*) [13].

Anti-TfR antibody and anti-insulin receptor antibody are clearly useful in applications requiring transport of large molecules across the BBB, and for increasing antibody concentrations in the brain. However, TfR and the insulin receptor are expressed not only in the vascular endothelial cells of the brain, but also in those of other organs, as well as in other cells types in, for instance, the liver [15]. These technologies therefore deliver drugs to tissues other than the brain. Consequently, anti-TfR antibody has a short half-life in the blood [11].

For this reason, the effectiveness of therapeutic antibodies that natively have a long half-life in the blood is significantly reduced if they bind to TfR. In order to increase the half-life of anti-TfR antibodies in the blood, some studies have attempted to reduce their binding strength or valence [14,16]. However, there remains a trade-off relationship for anti-TfR antibodies between high delivery ability into the brain and long half-life in the blood.

Therefore, we adopted a different approach that did not make use of the RMT concept. RMT enhances antibody transport across the BBB, but antibodies are also distributed in the body wherever antigens like TfR are expressed. We focused on slowing down antibody export from the brain, by binding the antibodies to a brain-specific antigen.

This antigen, myelin oligodendrocyte glycoprotein (MOG), which belongs to the immunoglobulin superfamily, is specifically expressed in oligodendrocytes. Oligodendrocytes exist only in brain and produce the myelin sheath, with MOG expressed on the outermost layer of the myelin. The molecular function of the protein is not well understood, but MOG-deficient mice present no clinical and histological abnormalities, and it is thought that MOG is an adhesion molecule [17–20].

Here, we report the use of an anti-MOG antibody to increase antibody and enzyme concentrations in the brain. We named this technology AccumuBrain. This new technology is expected to increase the levels of therapeutic proteins like antibodies or enzymes in the brain, and to maintain these levels for longer periods than technologies which use anti-TfR antibodies.

## Materials and methods

### Statement of compliance with ethical regulations and identity of the committee(s) approving the experiments

All animal studies were performed in accordance with Standards for Proper Conduct of Animal Experiments at Kyowa Hakko Kirin Co., Ltd. under the approval of the company’s Institutional Animal Care and Use Committee (protocol numbers APS14J0242, APS14J0286, APS15J0222, APS16J1050, APS16J1055, APS17J0153, and APS17J0155). Tokyo Research Park of Kyowa Hakko Kirin Co., Ltd. is fully accredited by the Association for the Assessment and Accreditation of Laboratory Animal Care, International.

### Animal models

Male ICR mice (*Mus musculus*) were purchased from Japan SLC (Japan) at 4 weeks of age. Sprague Dawley (SD) rats (*Rattus norvegicus*) were purchased from Charles River Japan (Japan) at 6 weeks of age. Mice and rats were housed in individually ventilated cages (IVC) with Paper-Clean chips (Japan SLC) and maintained in a specific pathogen-free (SPF) environment with a 12-hour light/dark cycle, controlled temperature and humidity, and free access to water and solid diet throughout the duration of the study.

After a quarantine/acclimation period of 1 week, mice were used at 5 weeks of age and rats were used at 7 weeks of age. During the quarantine/acclimation period, no animals were found to show abnormal clinical signs.

Animals were assigned to groups in an alternating manner, in order of decreasing body weights (heaviest to lightest) so that the variance between the groups was minimal. SD rats or ICR mice were intravenously injected with each antibody (see below). After the indicated time, rats or mice were perfused with 1% heparin-PBS (150 mL for rats and 6 mL/min for 8 min for mice) from the left ventricle, and their brains were extracted under anesthesia with pentobarbital. Blood was collected before perfusion. Brain weights were measured, after which brains were homogenized in citric buffer (pH 3.5). After centrifugation, the supernatant was isolated as a brain sample, and the volume was measured. Pellets were homogenized and eluted in citric buffer two more times (pH 3.5 and pH 2.5). Eluted samples were neutralized with 1 M tris-HCl (pH 9.0). Antibody concentrations were measured using an AlphaLISA kit (PerkinElmer). For each time point, three samples of antibody were eluted at pH values of 3.5, 3.5, and 2.5; the antibody concentration of each sample was then calculated (per gram brain), and the three antibody concentrations were added to determine the final concentration.

### Isolation of anti-MOG scFvs by Phage Display

Anti MOG single-chain variable fragments (scFvs) were isolated from naïve libraries using modified phage display protocols [21,22]. Briefly, the genes encoding the variable heavy (VH) and variable light (VL) chains were amplified by polymerase chain reaction (PCR) from human (*Homo sapiens*) peripheral blood mononuclear cell (PBMC) cDNA. PCR fragments of VH and VL were integrated into phagemid vector pCANTAB 5E (Amersham Pharmacia) and transformed into *Escherichia coli* TG1 (Lucigen, #60502–2). Purified plasmids and M13KO7 Helper Phage (Invitrogen) were used to construct an M13 phage naïve human scFv library. Rat MOG-FLAG Fc were immobilized to MAXISORP STARTUBE (NUNC) and blocked by SuperBlock Blocking Buffer (Thermo Fisher Scientific, #37515). The purified phage library was incubated for 1 h at room temperature in the plate. After washing with PBS containing 0.1% Tween 20 (PBS-T), phages binding to MOG were eluted with 0.1 M Gly-HCl (pH 2.2). Eluted phages were neutralized with Tris-HCl (pH 8.5) and used to infect TG1. Enriched phages were purified and incubated in the antigen-coated plate again. Two further rounds of antigen-binding selections were performed to enrich phage binding to MOG. Two phages binding to rat MOG-Fc were isolated, sequenced, and named MOG01 and MOG14.

### Production of anti-AVM antibody

Monoclonal anti-avermectin antibody was used as a negative control and was isolated by means of the standard hybridoma method [23]. Briefly, 4-week old SD rats were immunized four times with bovine serum albumin-avermectin (BSA-AVM). Aluminum hydroxide and *Bordetella pertussis* were added as adjuvants for the first immunization. The spleen was removed 3 days after the final immunization, and 1 × 10^8^ splenocytes were fused with 1 × 10^7^ P3-U1 cells in the presence of polyethylene glycol 1000 (Junsei, Tokyo, Japan). The screening of cultured hybridoma cells was performed using binding enzyme-linked immunosorbent assay (ELISA). From ELISA-positive hybridoma, cDNA was prepared, sequenced, and identified as anti-AVM antibody.

### Recombinant protein expression vector construction

For recombinant MOG01 and MOG14 production, DNA encoding the VH and VL of MOG01 or MOG14 and constant region of lambda light chain were cloned into N5KG4PE in which the human heavy chain IgG1 constant region was replaced with IgG4 with S228P and S235E mutations in N5KG1 [24]. MOG01 scFv and the hinge-CH2-CH3 region of human IgG4PE were amplified with PCR and cloned into N5KG4PE to create MOG01 scFv-Fc. As a negative control, VH and HV of rat anti-avermectin antibody were cloned into N5KG4PE. VH and VL of the anti-rat transferrin receptor antibody OX-26 [25] were cloned into N5KG4PE.

For symmetric bispecific antibody expression vector construction, the constant region of human IgG and MOG01scFv were amplified and assembled with PCR to construct CH1-Hinge-CH2-CH3-MOG01scFv, and then cloned into a pCI vector (Promega). Light chain and VH of the anti-AVM antibody were amplified with PCR and cloned into the MOG01 scFv expression vector. As a negative control, the constant regions of human IgG and anti-AVM scFv were amplified and assembled to construct CH1-Hinge-CH2-CH3-AVMscFv, with an inserted NheI-BamHI site in the anti-AVM antibody, and cloned into the MOG01 scFv expression vector.

For asymmetric bispecific antibody expression vector construction, an anti-AVM IgG4 antibody with S228P/S235E/R409K/S354C/T366W and an MOG01 IgG4 antibody with S228P/S235E/R409K/Y349C/T366S/L368A/Y407V were separately cloned into a pCI vector.

For enzyme fusion antibody expression vector construction, VH and VL of MOG01, the constant regions of lambda light chain and human IgG4 heavy chain were cloned into pCI vector (Promega). As a negative control, VH and HV of rat anti-avermectin antibody, constant region of kappa light chain and human IgG4 heavy chain were cloned into pCI vector. DNA coding for human acid sphingomyelinase (ASM) was cloned into the C-end of each antibody’s heavy chain.

For membrane-bound native MOG expression, full length DNA of human, rat, mouse, and cynomolgus monkey (*Macaca fascicularis*) MOG were cloned into pEF6/V5-His (Thermo Fisher Scientific). To construct the Fc-fusion protein, the extracellular domain of rat, mouse, and human MOG and FLAG tag-fused human Fc were cloned into INPEP4 (IDEC). To construct the GST-fusion protein, the extracellular domain of rat, mouse, and human MOG and GST were cloned into N5K (IDEC).

### Recombinant protein expression and purification

Vectors were infected into Expi293 using the Expi293 Expression System (Thermo Fisher Scientific) for recombinant protein expression. Antibodies and Fc-fusion proteins were purified with ProteinA (MabSelect SuRe, GE Healthcare), and were eluted with citrate buffer (20 mM of sodium citrate and 50 mM NaCl, pH 3.4) and neutralized with 1 M Tris-HCl buffer (pH 8.0). GST-fusion proteins were purified using Glutathione Sepharose 4B (GE Healthcare), and eluted with glutathione buffer (50 mM of Tris-HCl and 10 mM of reduced glutathione, pH 8.0). After elution, samples were ultrafiltrated with VIVASPIN (Sartorius) and buffer was exchanged with PBS using a NAP Column (GE Healthcare).

### ELISA

For ELISA, rat MOG-FLAG Fc were immobilized to MAXISORP STARTUBE (NUNC) and blocked by SuperBlock Blocking Buffer (Thermo Fisher Scientific, #37515). As a negative control, FLAG Fc immobilized plates were prepared. Purified phage clones were incubated in these plates for 30 min at room temperature. After washing with PBS-T, PBS-T with HRP-labeled anti-M13 antibody (GE Healthcare) and 10% BlockAce (Dainippon, #UKB40) were added and incubation was carried out for 30 minutes at room temperature. After washing thrice with PBS-T, TMB solution (DAKO) was added and a further incubation was performed for 30 minutes at room temperature. After adding 0.5 M sulfuric acid to stop the reaction, the absorbance was measured at a wavelength of 450 nm/570 nm using a microplate reader (Molecular Devices).

### Flow cytometry

The binding activity of anti-MOG antibody was determined by flow cytometry. Anti-AVM antibody was used as a negative control. Membrane-bound rat, mouse, cynomolgus monkey, and human MOG expression vectors were used to infect Expi293 cells using the FreeStyle 293 Expression System (Thermo Fisher Scientific). Cells were suspended in 96-well round-bottomed plates at a density of 5×10^5^ cells/well and washed with FACS buffer (1% BSA, 2 mM EDTA and 0.1% sodium azide in D-PBS). Each of the MOG antibodies and the rat anti-AVM antibody were added to wells at concentrations of 10 μg mL^-1^ at 4°C for 30 minutes. After washing, secondary antibody (RPE-labeled Goat Anti-Human IgG Antibody, Southern Bioblot) was added to each well at concentrations of 1 μg mL^-1^ and incubated at 4°C for 30 minutes. After washing, the cells were analyzed using a FACS Canto II System (BD Biosciences).

### Kinetic Analysis of anti-MOG antibody binding properties using Biacore

Binding affinity and kinetics analyses were conducted using a Biacore T100 optical biosensor and a Human Antibody Capture Kit (GE Healthcare, Piscataway, NJ). The murine anti-human IgG antibody was immobilized on a CM5 sensor chip according to the manufacturer’s instructions. MOG antibodies were captured as ligands, and rat, mouse, cynomolgus monkey, and human MOG-GST fusion proteins were passed over the captured antibodies as analytes. Binding kinetic parameters, including the ka, kd, and KD values, were calculated using Biacore evaluation software.

### IVIS test

Anti-MOG antibody and anti-AVM antibody were labeled with an Alexa FluorR 488 Protein Labeling Kit (Molecular Probes). Mice were intravenously injected with AF488-labeled anti-MOG antibodies and control anti-AVM antibody (10 mg kg^-1^). After the indicated time, tomato lectin was injected into mice and blood was collected. On the same day, mice were perfused with PBS and their brains were extracted under anesthesia with pentobarbital. Fluorescence intensity of brains and blood were measured using an IVIS Spectrum system (Perkinelmer).

### Statistical analysis

All values are expressed as the mean ± S.D. Statistical analyses were performed using a statistical analysis software program (SAS, release 9.2; SAS Institute, Inc., Cary, NC, USA). Differences between the control antibody group and the anti-MOG or anti-TfR antibody group were assessed using Student’s *t*-test or Aspin–Welch test. Differences were considered to be statistically significant at a *P*-value of < 0.05.

## Results

### Preparation of human/monkey/mouse/rat cross-reactive anti-MOG antibody

**Fig 1:**
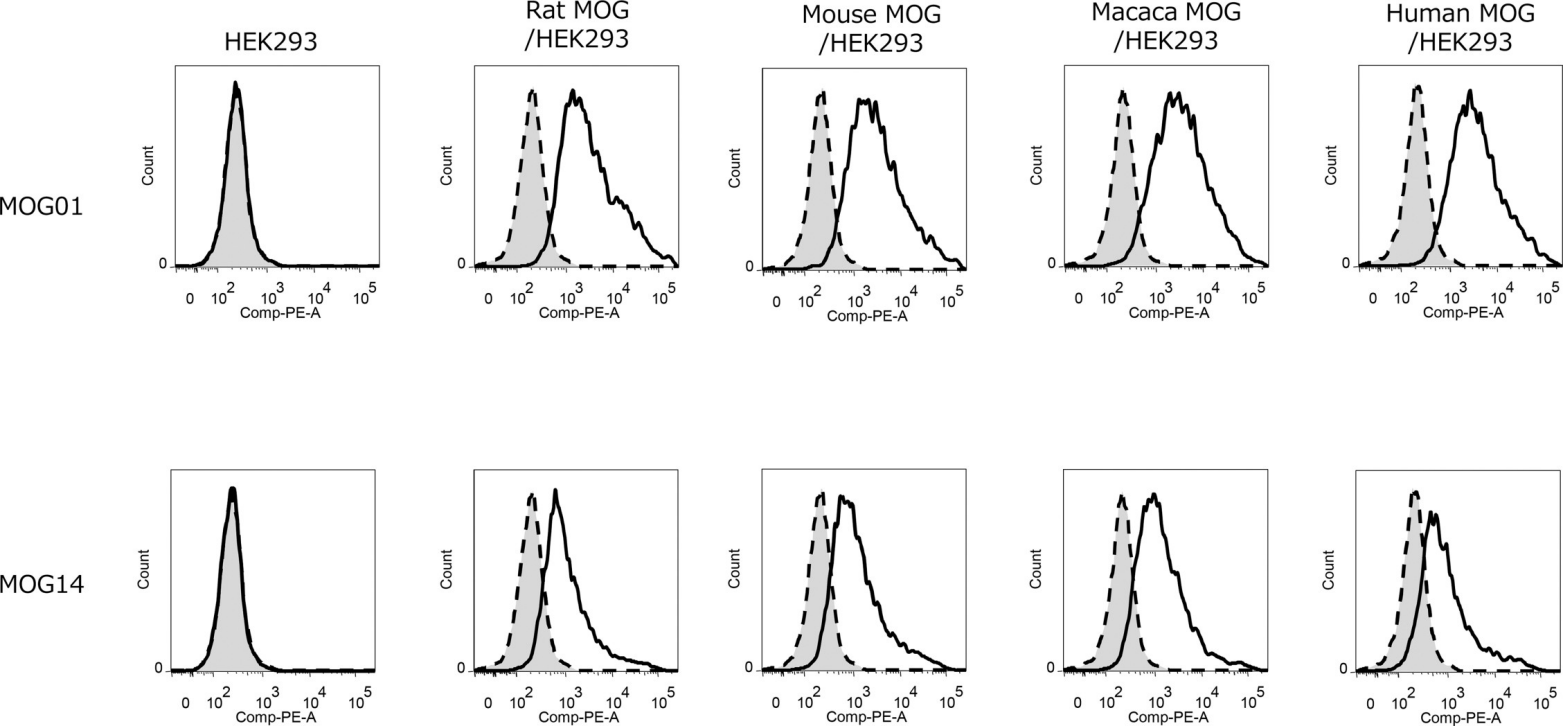
Cross-reactivity of anti-MOG antibodies. Histograms of anti-MOG antibodies binding to rat, mouse, cynomolgus, and human MOG expressed in HEK 293 cells as determined by flow cytometry (solid line). Anti-AVM antibody was used as a negative control (broken line). Anti-MOG antibodies MOG01 and MOG14, and rat anti-AVM antibody as a negative control, were added at concentrations of 10 μg mL^-1^. Antigen binding antibody was detected by an RPE-labeled goat anti-human antibody (Southern Bioblot).

At first, we obtained human anti-MOG antibodies that were cross-reactive among human, monkey, mouse, and rat MOG. This cross-reactivity is required for use in clinical, primary safety testing, and experimental animal models (sequence arrangement is in S1 Fig). An M13 phage naïve human scFv library was constructed from human PBMC derived cDNA. Anti-MOG antibodies were screened with rat MOG-Fc fusion protein from this library, and we obtained two anti-MOG antibodies, MOG01 and MOG14, capable of binding human, monkey, mouse, and rat MOG (Fig 1). We confirmed MOG01 cross-reactivity by means of Biacore analysis (Table 1).

**Table 1:** Biacore analysis of anti-MOG antibody.

Ligand	Analyte	ka (1/Ms)	kd (1/s)	KD (M)	Rmax (RU)	U-value	
MOG01 scFv-Fc	Rat MOG	1.8E+06	4.8E-03	2.60E-09	43	2	
	Mouse MOG	1.7E+06	8.9E-03	5.14E-09	43	2	
	Cyno MOG	1.2E+06	7.1E-03	5.98E-09	55	1	
	Human MOG	9.9E+06	4.1E-02	4.11E-09	62	20	※#

※ Kinetic constant ka is approaching the limit that can be measured by the instrument.

# Kinetic constants were difficult to determine.

MOG: myelin oligodendrocyte glycoprotein KD: dissociation constant RU: Resounance Unit

### Accumulation of anti-MOG antibodies in rat brains and comparison with anti-TfR antibody

Anti-MOG antibodies were injected into SD rats *via* the tail vein. Anti-AVM antibody was used as a negative control. On day 4, rats were perfused and their brains extracted, homogenized, and processed to recover the antibodies they contained. Antibody concentrations in the plasma was identical in animals treated with anti-MOG antibodies and the negative control. The concentration of anti-MOG antibody in the brain was 5–10 times higher than in the negative control (S2 Fig). MOG01 was therefore selected for use in the following experiments.

There are many studies which report that anti-TfR antibodies are increased in the brain through RMT. However, there are also reports that anti-TfR antibodies have shorter half-lives in plasma. Therefore, we compared differences in plasma and brain concentrations of anti-MOG antibody MOG01 and an anti-TfR antibody (OX-26) which has been reported to increase antibody concentration in the brain of rats [26].

**Fig 2:**
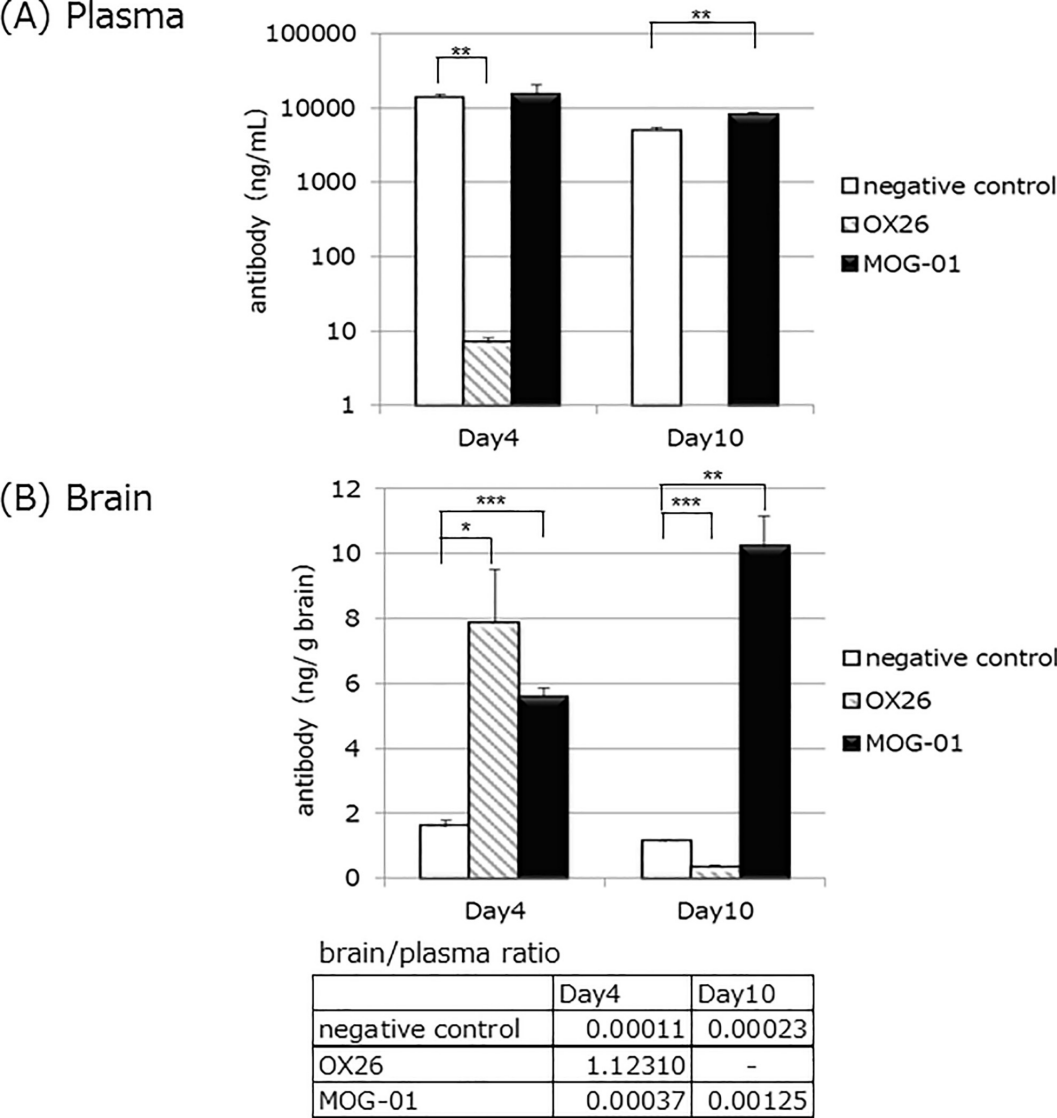
Comparison of anti-TfR antibody OX-26 and anti-MOG antibody MOG01 concentration in plasma and brain. Rats were injected *via* the tail vein with negative control (white bar, n = 3), anti-TfR antibody OX-26 (hatched bar) or anti-MOG antibody MOG01 (black bar). At days 4 and 10, rats were perfused with PBS and their brains were extracted, the weights measured, homogenized, and eluted in citric buffer for antibody recovery. Blood was collected prior to perfusion from the tail vein. (A) Antibody concentration in the plasma (B) antibody amount, μg g^-1^ brain. Data represent the mean ± SD. *P<0.05, **P<0.01, and ***P<0.001.

Anti-MOG antibody MOG01, anti-rat TfR antibody, OX-26 and negative control anti-AVM antibody were injected into SD rats (n = 3 for each time point) *via* the tail vein (5 mg kg^-1^, Fig 2). OX-26 showed a dramatic decrease in plasma level at day 4, which further decreased to below the detection limit at day 10. In contrast, the plasma concentration of MOG01 antibody was the same as that of the control antibody on both day 4 and day 10 (Fig 2A).

On day 4, OX-26 antibody concentrations in the brain were increased, as previously reported. However, on day 10, OX-26 levels were lower than those of the control antibody. In contrast, the concentration of MOG01 in the brain was higher than that of the control antibody, and comparable to that of OX-26, on day 4. On day 10, MOG01’s concentration in the brain was much higher than that of OX-26 and the control antibody, and also than its own level at day 4 (Fig 2B).

### Anti-MOG antibody concentrations in mouse brains remain high for extended periods

**Fig 3:**
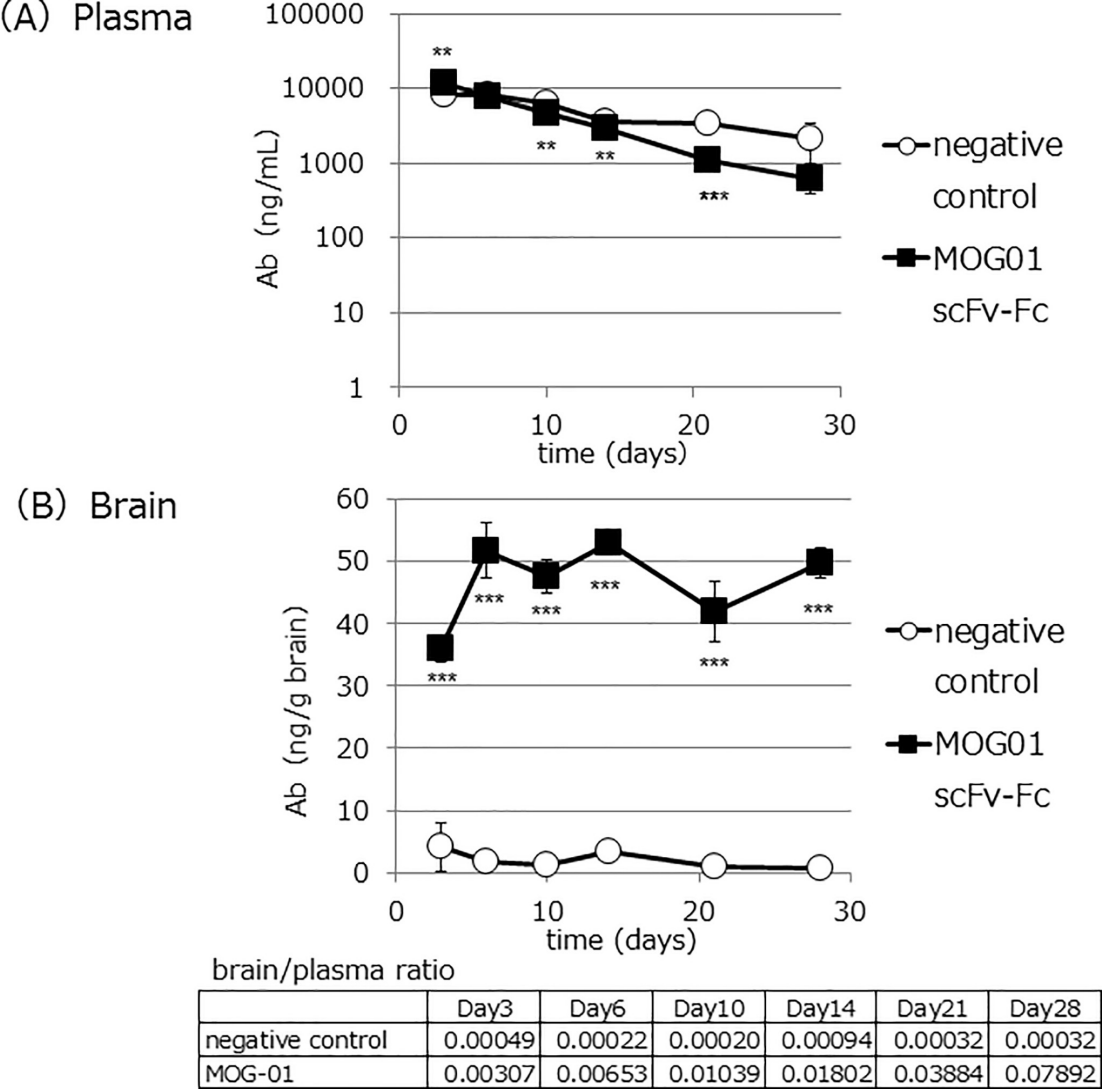
Long-term brain accumulation of anti-MOG antibody. Pharmacokinetic evaluation of peripheral and brain antibody level following intravenous injection of the negative control and of anti-MOG antibody MOG01. Mice were intravenously injected with anti-MOG antibody and control anti-AVM antibody (5 mg kg^-1^, n = 3 for each time point). After the times indicated, mice were perfused with PBS and their brains were extracted. The brain weights were measured, after which they were homogenized and eluted in citric buffer for antibody recovery. Blood was collected prior to perfusion from the tail vein. (A) Antibody concentration in the blood and (B) in the brain of MOG01 (black square) and negative control (white circle). Data represent the mean ± SD. **P<0.01 and ***P<0.001.

Next, we checked antibody concentration changes over a long term in mice. MOG01 scFv-Fc was injected into mice (n = 3 for each time point) *via* the tail vein 5 mg kg^-1^). Brains and plasma were collected on days 3, 6, 10, 14, 21, and 28 (Fig 3). Antibody concentrations in plasma of MOG01 scFv-Fc were comparable to those of the control antibody on days 3 to 14, and were lower on days 21 and 28 (Fig 3A). In contrast, MOG01 antibody concentrations in mouse brains were dramatically increased on day 3, and these levels were maintained for one month (Fig 3B). This data suggested that use of the anti-MOG antibody allows for high antibody concentrations in the brain to be maintained for at least one month, extending the dosing interval.

### IVIS imaging of anti-MOG antibody in mouse brains

**Fig 4:**
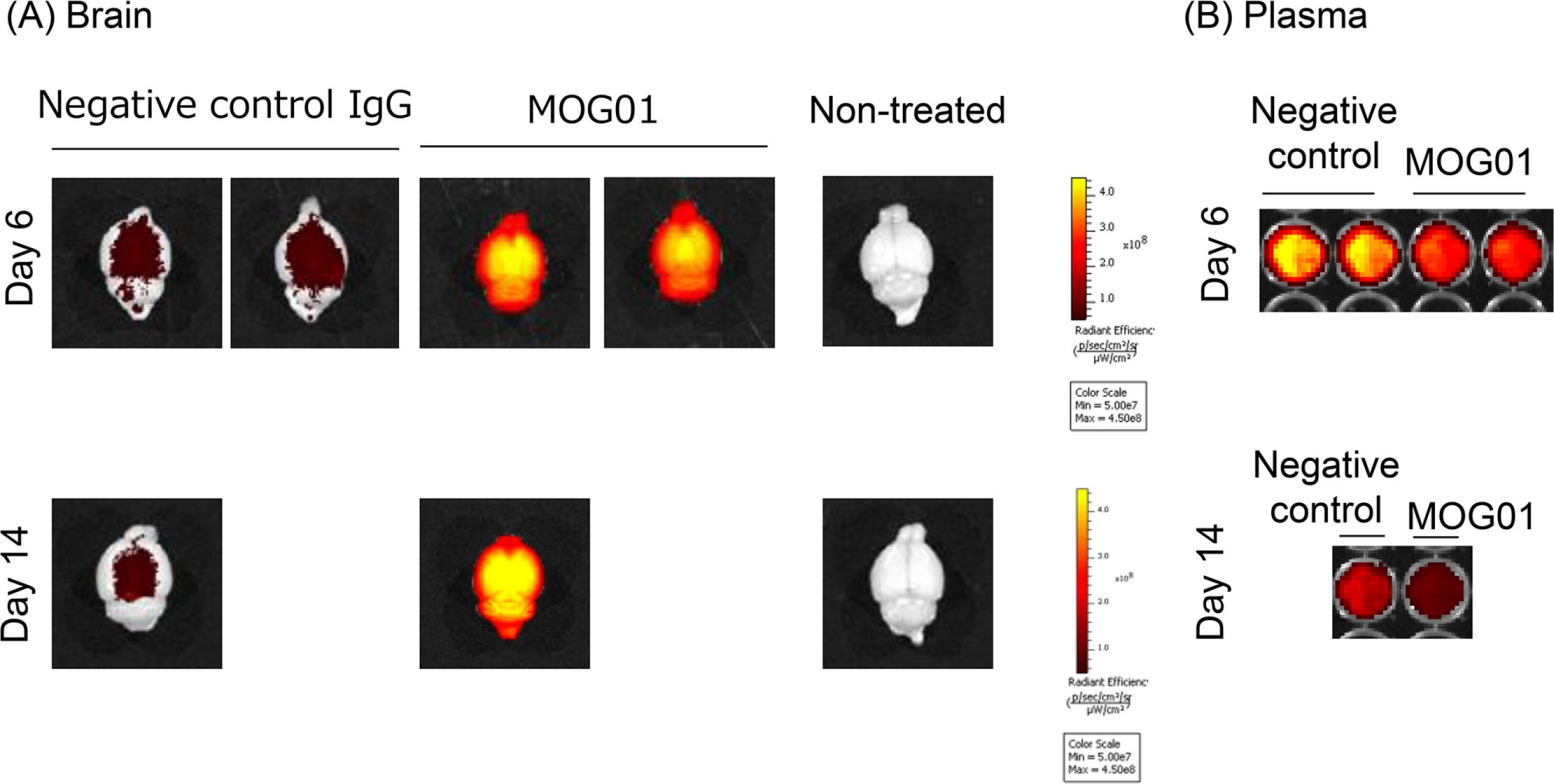
IVIS imaging of anti-MOG antibody in the brain. Anti-MOG antibody MOG01 and anti-AVM antibody as negative control were labeled using an Alexa Fluor 488 Protein Labeling Kit (Molecular Probes). Labeled antibodies were injected into mice *via* the tail vein (10 mg kg^-1^). After the indicated time, mice were perfused with PBS and their brains were extracted. The fluorescence strength of each brain was then measured. IVIS imaging of brain (A) and in plasma (B) at days 6 and 14.

For IVIS imaging, MOG01 and the appropriate control antibody were labeled with Alexa FluorR 488 and injected into mice *via* the tail vein (10 mg kg^-1^). On day 6 or day 10, mice were perfused, their brains were extracted, and the fluorescence intensity of each brain was measured with IVIS (Fig 4). Labeled MOG01 showed higher fluorescence intensity than that of the control antibody, whereas the fluorescence intensity of MOG01 in plasma was lower than that of the control. These results indicated that MOG01 accumulated in the brain, showing a much higher brain/plasma ratio. Fluorescence of MOG01 did not appear to be site-specific, but was spread throughout the brain, although MOG is expressed specifically in the myelin sheath and the anti-MOG antibody was therefore expected to accumulate there.

### Delivery of antibodies or enzymes linked to anti-MOG antibodies to mouse brains

**Fig 5:**
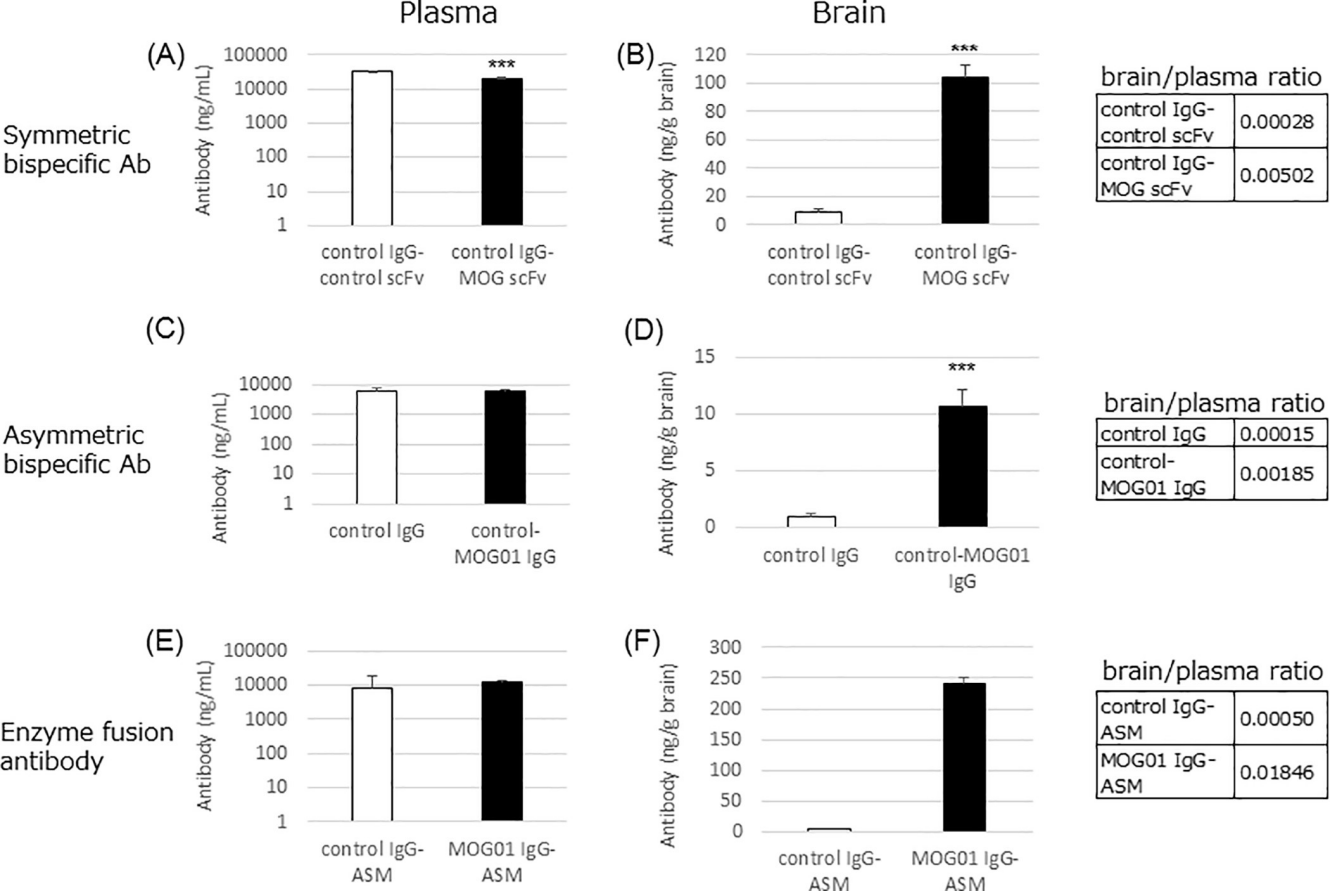
Brain accumulation of bispecific antibody and antibody-enzyme fusion protein. Mice were injected *via* the tail vein with control antibody fused with control scFv as a control, or with anti-MOG scFv (5 mg kg^-1^, n = 3). On day 10, bispecific antibodies were collected from the plasma and the brain. Bispecific antibody concentration in the blood (A) and in the brain (B) of control-control bispecific antibody (white bar) and control-MOG bispecific antibody (black bar). Asymmetric bispecific antibody control-MOG01 IgG (black bar) was tested (5 mg kg^-1^, n = 4), and concentrations were measured in the blood (C) and in the brain (D). Acid sphingomyelinase (ASM) fused with anti-MOG antibody and control antibody fused with ASM as a control were injected into mice *via* the tail vein (n = 4). On day 10, antibodies fused to ASM were collected from the plasma and the brain. Antibody-enzyme concentration in the blood (E) and in the brain (F) of mice injected with control (white bar) and MOG (black bar) antibody-enzyme fusion protein. In the brain, 2 of 4 control samples were below the limit of detection. Data represent the mean ± SD. ***P<0.001.

We evaluated the applicability of anti-MOG antibodies for therapeutic use (Fig 5). We generated two bispecific antibody formats (S3 Fig). The first was a symmetric bispecific antibody created by fusing anti-MOG scFv to a control IgG at the C end of the Fc region. A control antibody fused with control scFv was also generated. The second was an asymmetric bispecific antibody similar to IgG, but which had a different Fab as a result of asymmetrical Fc engineering to generate a hetero heavy chain assembly. These bispecific antibodies were injected into mice, and their brains and plasma were collected on day 10. The concentrations of bispecific antibodies in plasma were comparable to those of the respective control antibodies (Fig 5A & 5C). In contrast, the MOG01 bispecific antibody levels were about 10-times higher than those of the control antibodies in the brain (Fig 5B & 5D). These results suggested anti-MOG antibodies increase therapeutic antibody concentrations in the brain, independent of the bispecific antibody format.

Finally, we generated an anti-MOG antibody fused with enzyme acid sphingomyelinase (ASM) at the C end of the Fc region. ASM is the enzyme responsible for lysosomal storage disorders such as Niemann-Pick disease. Enzyme-fused antibodies were injected into mice (n = 4) *via* the tail vein 5 mg kg^-1^), and mouse brains and plasma were collected on days 10 and 28. Two of four mice injected with ASM fused to the control antibody displayed brain enzyme-fused antibody concentrations below the lowest limit of detection. ASM fused with MOG01 was present at much higher levels in the brain than was ASM fused with the control antibody.

## Discussion

Many antibody therapeutics for oncological and autoimmune diseases have either already been launched, or are currently undergoing clinical trials. Similarly, many therapeutic antibodies for brain and CNS diseases are in clinical trial, but some of these are required to be injected in large quantities, and few have thus far been approved. It is well known that the concentration of proteins, including therapeutic antibodies, in the brain is very low when they are administered either intravenously or subcutaneously. The reason for this is that the BBB blocks the penetration of therapeutic antibodies from plasma into the brain. It has also been reported that antibodies are rapidly discharged from brain by FcRn.

There are many challenges to increase antibody concentration in the brain. RMT is currently the most popular mechanism for delivery of antibodies into the brain. TfR and insulin receptor show transcytosis from blood to brain, and binding molecules to these receptors can be delivered into brain by RMT. But these receptors are widely expressed in the body, and the plasma half-life of the antibodies are therefore decreased. In our study, we showed that anti-TfR antibody OX-26 was present at a high concentration in the brain on day 4 but that it decreased rapidly in the plasma. As a result, its antibody concentration was no longer detected by day 10. A anti-TfR antibody modified to overcome its short plasma half-life has been reported, but this antibody was retained for only one week [11].

Antibodies for therapeutic use typically have a long half-life in blood, and are usually administered once every other week or every month. In the case of TfR antibody-based technologies, more frequent administration, or higher administered amounts, are needed. In enzyme replacement therapy for lysosome diseases, therapeutic enzymes are usually also administered once every other week. Therefore, any brain-targeting technology involving therapeutic proteins would require less frequent administration.

Here, we established a new technology named AccumuBrain, which allows antibody concentrations in the brain to be greatly increased (ten times or more) and maintained at these levels for more than one month. In AccumuBrain, antibodies bind to MOG, the expression of which is myelin-specific, making the system central nervous system-specific. This means that the antibody concentration is increased only in the brain, and is decreased to a minimal level in plasma. This low level allows the antibody to maintain a long half-life in plasma, while increasing the brain concentration over an extended period.

It has been reported that anti-MOG autoantibodies were detected in the brain of multiple sclerosis (MS) and neuromyelitis optica (NMO) patients. One reason for this may be that anti-MOG autoantibody-expressing B cells have been detected in the brain [27, 28]. Another reason might be that in these patients, the tight junctions of the BBB might be looser, thereby increasing autoantibody permeability [29,30]. It was also reported that co-injection of CNS-specific T cells and anti-MOG antibodies led to the demyelination of CNS neuron cells [31]. In this report, activated T cells were recruited to the CNS-mediated BBB opening, and thus antibodies were allowed to pass through the BBB. Conversely, it is assumed that the antibody concentration in the brain does not increase under immune-inactivated conditions, and there are also reports that the anti-MOG antibody itself does not induce immune activation [32,33]. In addition, we used a mutated anti-MOG IgG4 antibody in which the effector functions have been inactivated [34]. These suggest that anti-MOG antibody does not open the BBB via immune activation. We assume that our anti-MOG antibodies pass through the BBB in the same manner as the control antibody but that their efflux from the brain is slowed by binding to MOG, resulting in higher antibody concentrations in the brain. However, this mechanism is still hypothetical, and further analysis is needed in the future.

TfR is expressed in brain vessels. Anti-TfR antibody binds to TfR and is then delivered into the brain by RMT. However, using the whole brain collection method, it is difficult to determine whether the anti-TfR antibody is delivered into brain, or whether it remains in the blood. It has been reported that a certain amount of anti-TfR antibody binds directly to brain vessels [16]. Whole brain samples would therefore include some amount of antibody that bound to brain vessels on the blood side, which could result in overestimation of the amount of TfR antibody in the brain. On the other hand, MOG is expressed at the myelin, which is brain specific, and so it is expected that anti-MOG antibody would be increased in the brain, but not in brain vessels. Our IVIS data suggested that antibodies were increased across the whole brain, and not in a site-specific manner.

From the results of the long-term testing, the plasma concentration of MOG01 was found to be decreased. This could be due to antigen-dependent clearance, and some of the antibody is thought to be captured and internalized by oligodendrocytes.

For therapeutic applications, safety testing in monkeys and evaluation of drug efficacy in rodent models are essential. We developed anti-MOG antibodies that can bind human, monkey, rat, and mouse antigen at the same level, which will enable us to perform accurate evaluations in future preclinical studies.

Safety issues are a concern when using anti-MOG antibodies in therapeutic applications. MOG peptide, or MOG itself, are known to be capable of inducing experimental allergic encephalomyelitis (EAE) in mouse models [35]. On the other hand, there are reports that the MOG antibody is not itself pathogenic [32,33], meaning that not all anti-MOG antibodies would be pathogenic. Complement-dependent cytotoxicity (CDC) is induced by IgG1 and IgG3 and important for the pathogenicity of anti-MOG antibodies. An IgG1 subclass of anti-MOG antibodies is detected in MS and NMO patients [36,37]. Human IgG4 does not show CDC [38] and substitution of glutamic acid for serine at position 235 in the CH2 domain of IgG4 prevents Fc receptor binding [34]. These reports suggest that an engineered human IgG4 format would reduce the safety risk. Further investigations into MOG antibody’s safety for therapeutic use are needed.

In conclusion, we established a new technology, named AccumuBrain, which allows increased antibody concentrations of >10-fold to be achieved in the brain over an extended period of time. The long-term presence of antibodies made possible by the AccumuBrain technology is promising as a significant improvement over RMT-based technologies, e.g. anti-TfR antibodies. Our technology may constitute a breakthrough in the therapeutic protein targeting of central nervous system diseases.

## Supporting information

S1 Fig Alignment of mouse, rat, monkey, and human MOG amino acid sequence. (TIF)

S2 Fig In vivo accumulation of anti-MOG antibodies in brain. Rats were injected *via* the tail vein with negative control (white bar, n = 2) or anti-MOG antibodies (black bar). On day 4, rats were perfused with PBS and their brains were extracted and the weights measured, after which they were homogenized and eluted in citric buffer for antibody recovery. Blood was collected prior to perfusion from the tail vein. (A) Antibody concentration in the plasma (B) antibody amount, μg gram^-1^ brain.
(TIF)

S3 Fig Structure of bispecific antibodies. (TIF)

S1 Dataset Dataset for Fig 2. (TIF)

S2 Dataset Dataset for Fig 3. (TIF)

S3 Dataset Dataset for Fig 3. (TIF)

S4 Dataset Dataset for Fig 5. (TIF)

S5 Dataset Dataset for Fig 5. (TIF)

S6 Dataset Dataset for Fig 5. (TIF)
