# Supplementary figures and images for: A new technology for increasing therapeutic protein levels in the brain over extended periods
Source: PLoS One. 2019 Apr 12;14(4):e0214404. doi: 10.1371/journal.pone.0214404 (PMC6461266; doi:10.1371/journal.pone.0214404)

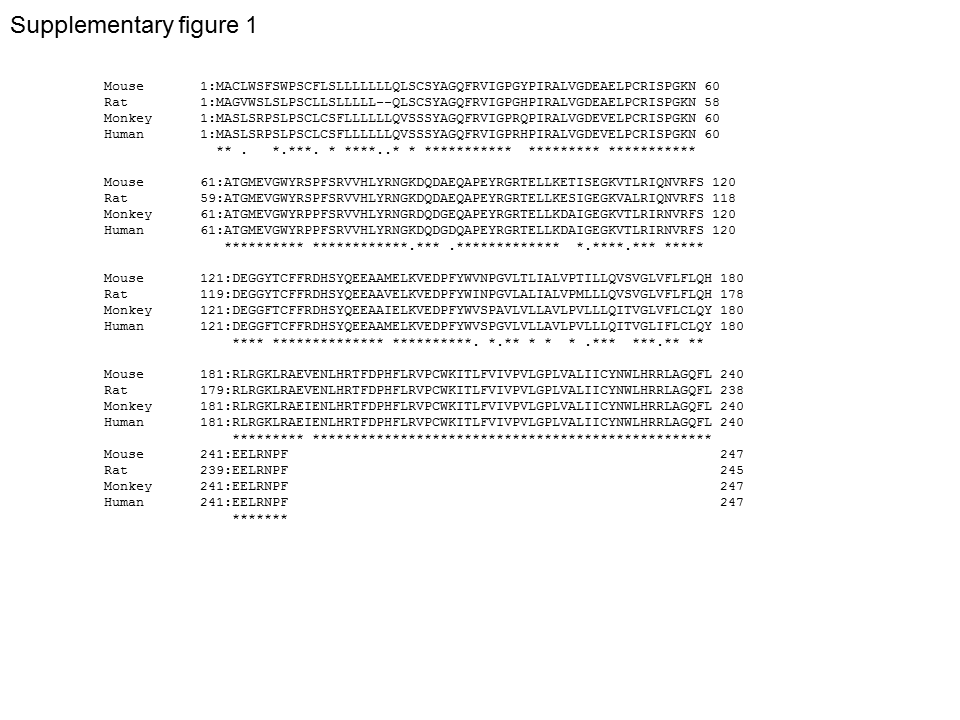

Supplement: S1 Fig — (TIF) [file pone.0214404.s001.tif]

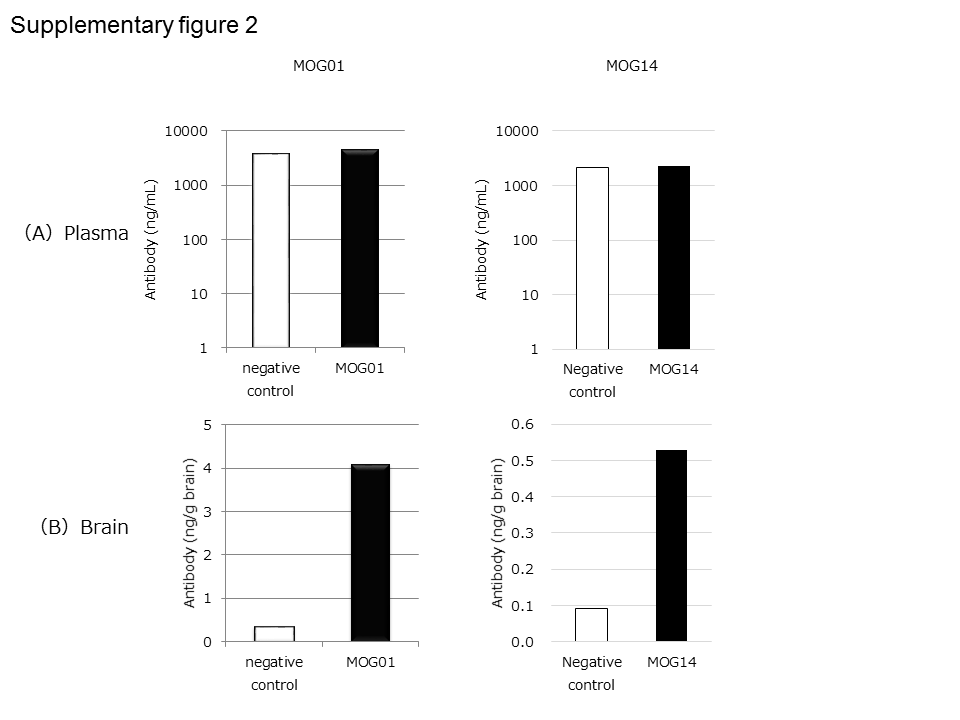

Supplement: S2 Fig — Rats were injected via the tail vein with negative control (white bar, n = 2) or anti-MOG antibodies (black bar). On day 4, rats were perfused with PBS and their brains were extracted and the weights measured, after which they were homogenized and eluted in citric buffer for antibody recovery. Blood was collected prior to perfusion from the tail vein. (A) Antibody concentration in the plasma (B) antibody amount, μg gram-1 brain. (TIF) [file pone.0214404.s002.tif]

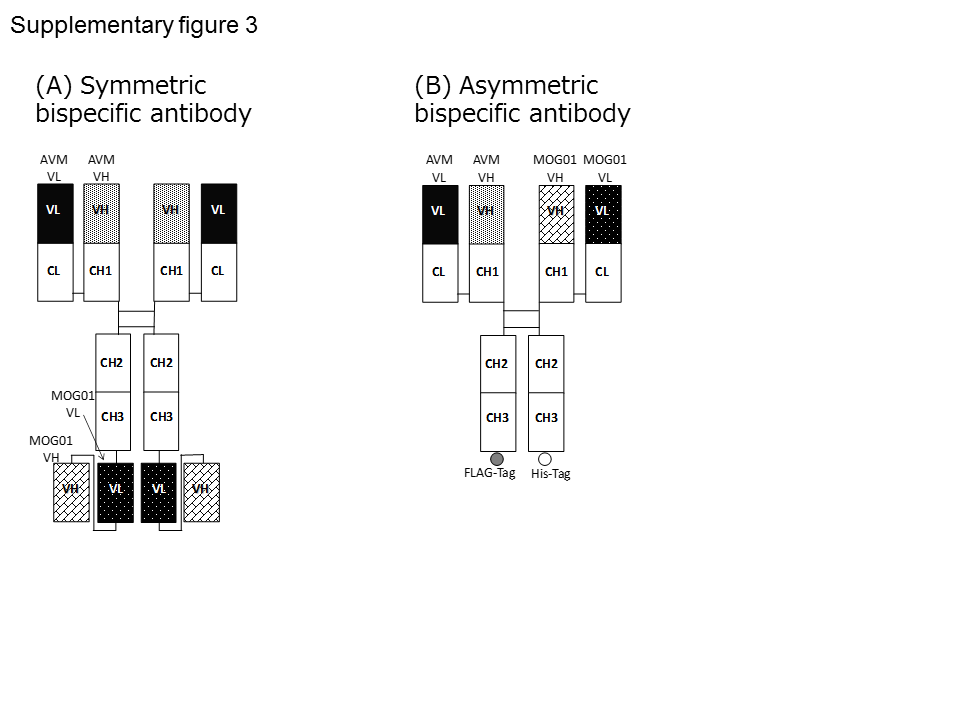

Supplement: S3 Fig — (TIF) [file pone.0214404.s003.tif]

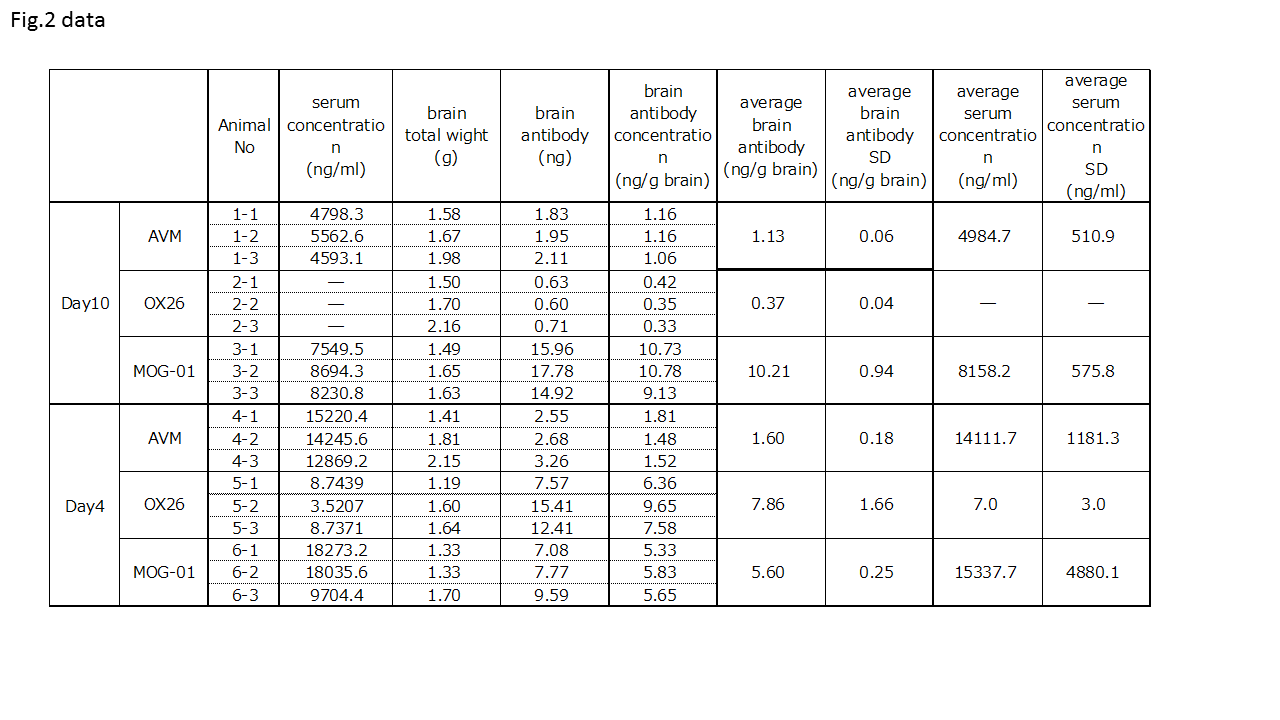

Supplement: S1 Dataset — (TIF) [file pone.0214404.s004.TIF]

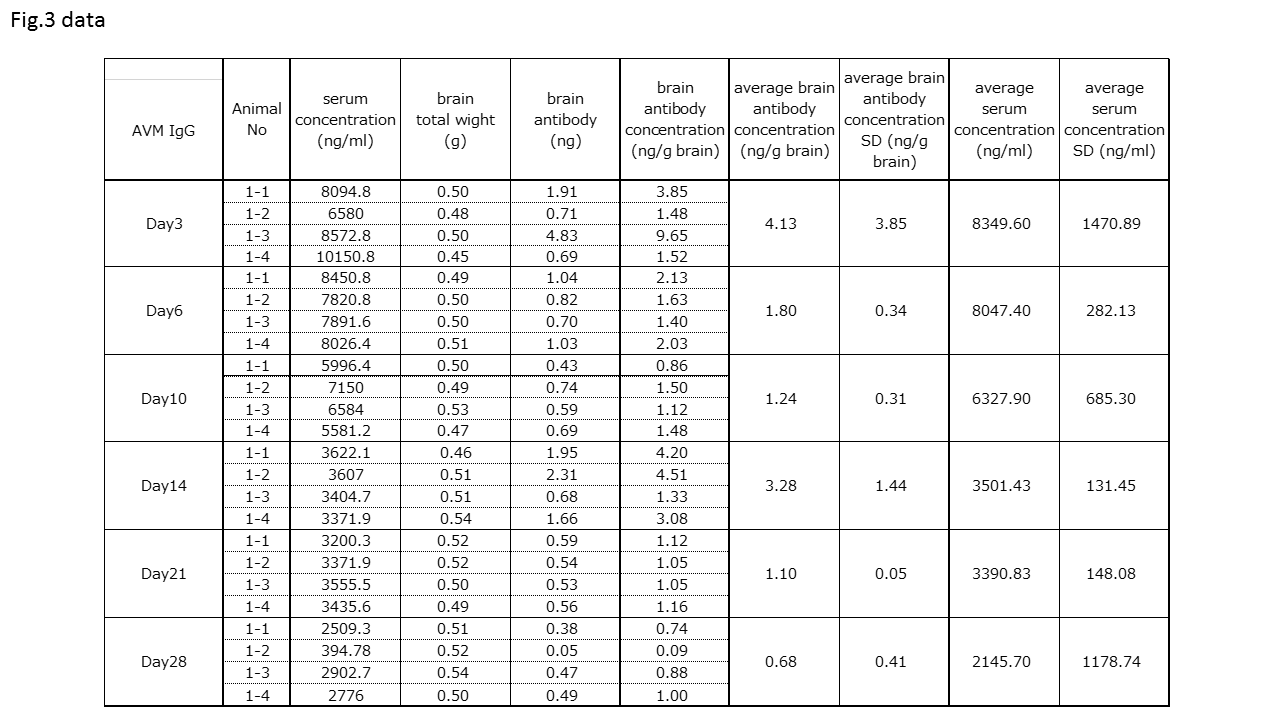

Supplement: S2 Dataset — (TIF) [file pone.0214404.s005.TIF]

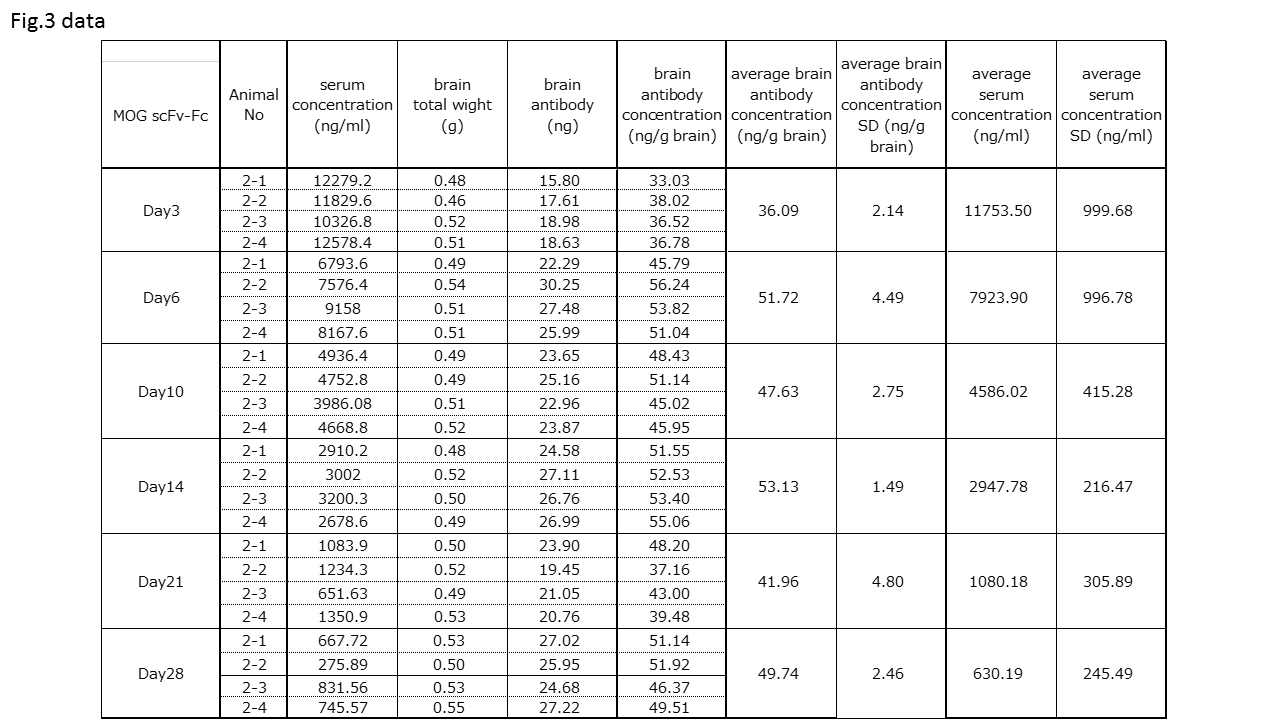

Supplement: S3 Dataset — (TIF) [file pone.0214404.s006.TIF]

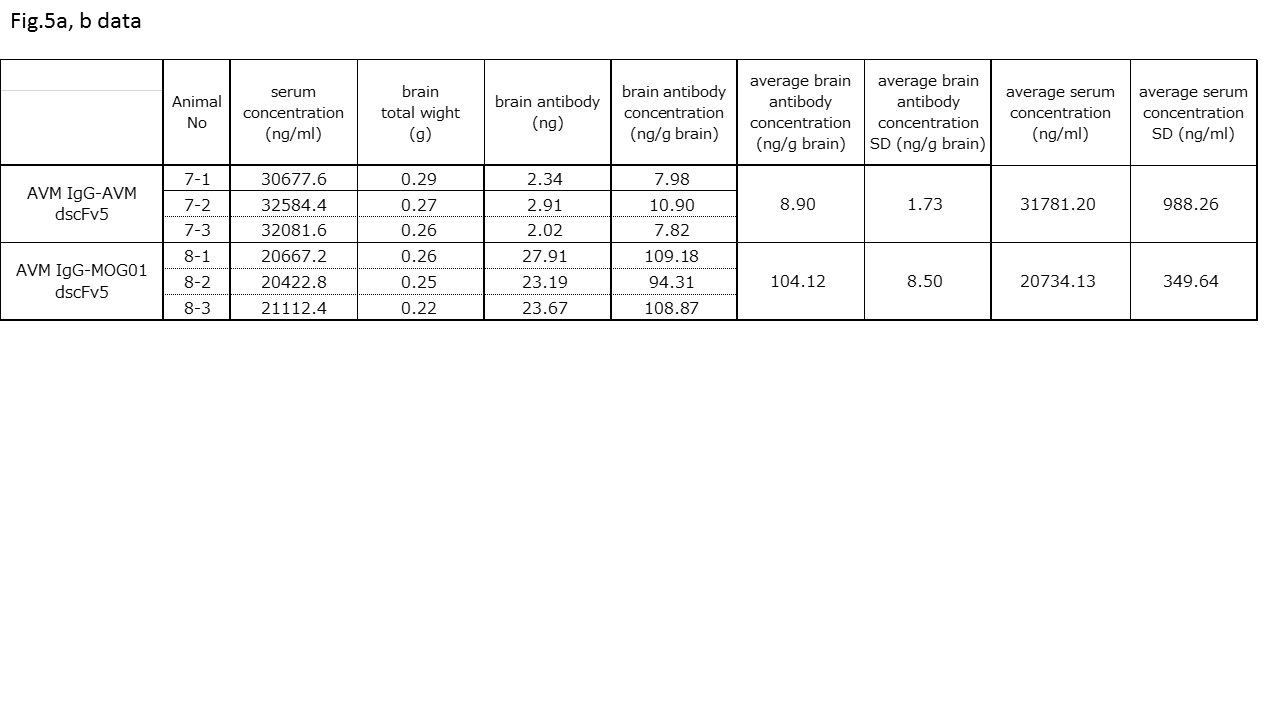

Supplement: S4 Dataset — (TIF) [file pone.0214404.s007.TIF]

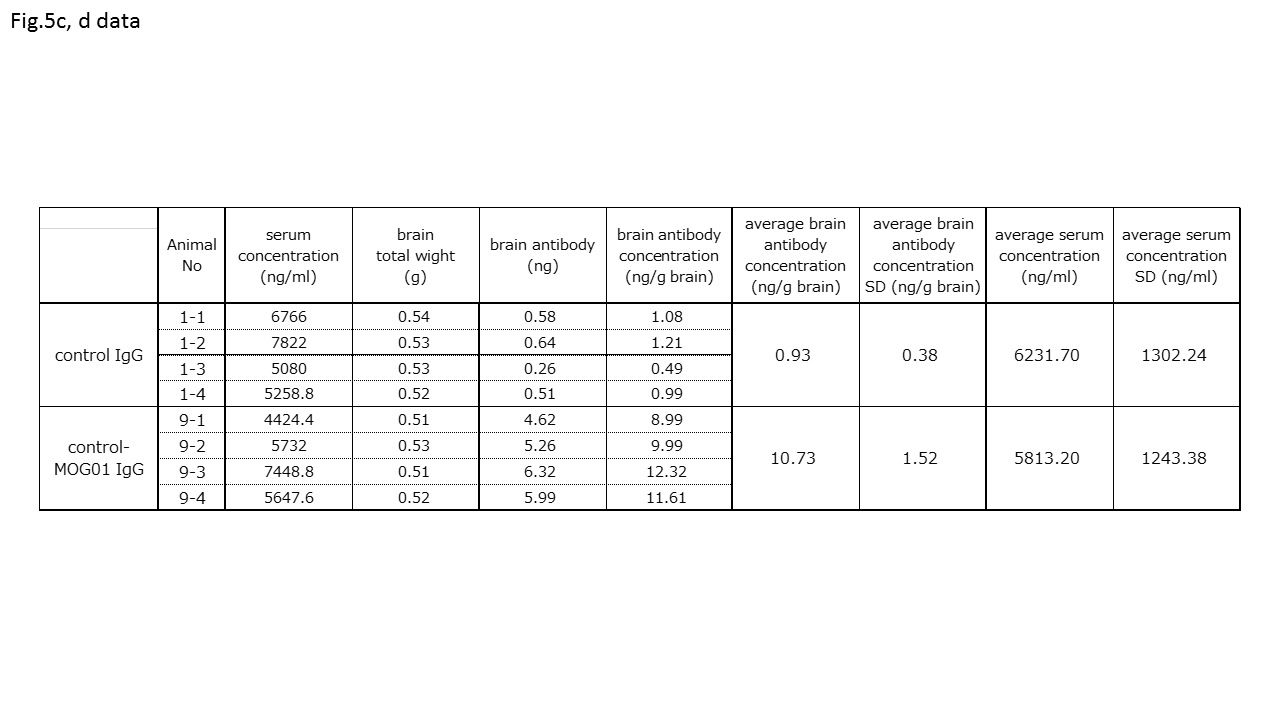

Supplement: S5 Dataset — (TIF) [file pone.0214404.s008.TIF]

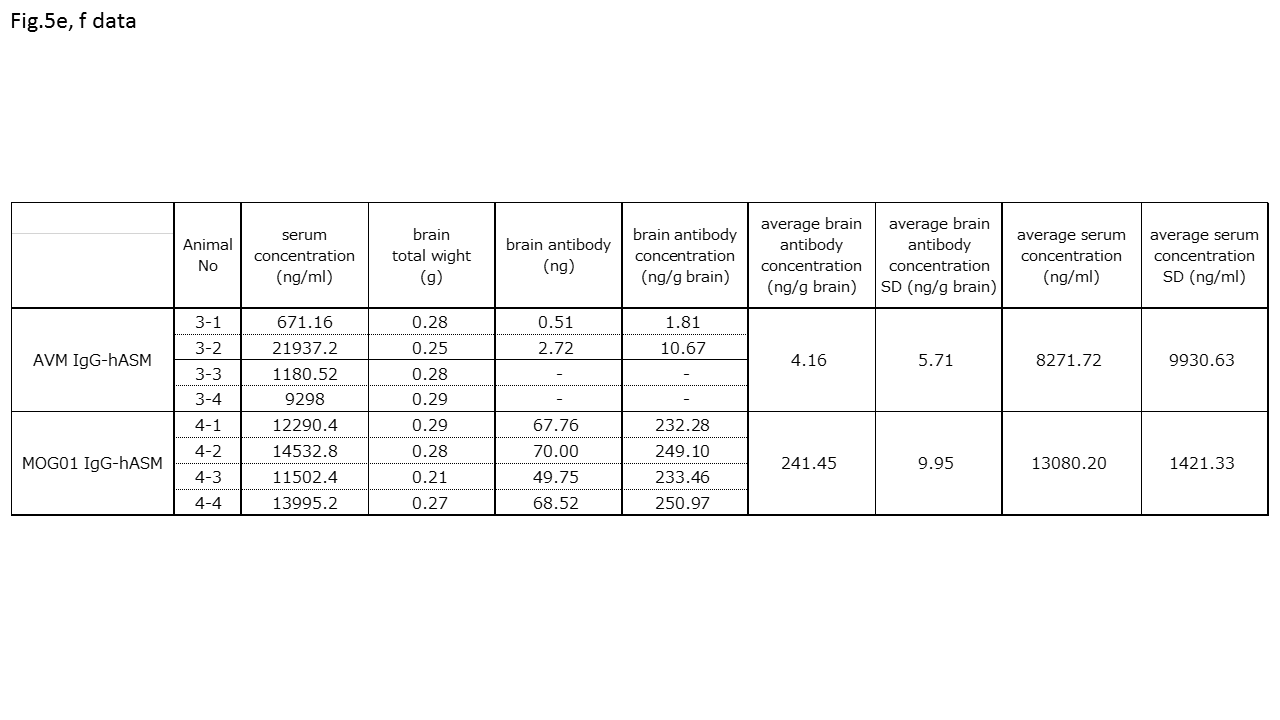

Supplement: S6 Dataset — (TIF) [file pone.0214404.s009.TIF]
